# Sol–gel engineered xanthan gum/carboxymethyl cellulose/silica hybrid adsorbent for efficient removal of cationic and anionic dyes

**DOI:** 10.1039/d6ra05243f

**Published:** 2026-07-15

**Authors:** Ehab S. Gad, Tayel A. Al Hujran, Saad Alrashdi, Mohammed A. Amin, Medhat E. Owda, Yousef A. Bin Jardan

**Affiliations:** a Department of Chemistry, College of Science, Jouf University Sakaka 72341 Aljouf Saudi Arabia; b Department of Pharmaceutical Chemistry, Faculty of Pharmacy, Mutah University Jordan; c Department of Pharmaceutics, College of Pharmacy, Qassim University Qassim 51452 Saudi Arabia; d Department of Chemistry, Faculty of Science (boys), Al-Azhar University 11884 Nasr City Cairo Egypt medhatowda@azhar.edu.eg; e Department of Pharmaceutics, College of Pharmacy, King Saud University Riyadh Saudi Arabia

## Abstract

Synthetic dyes released from industrial activities continue to pose a serious threat to aquatic systems, and their treatment requires adsorbents that are efficient, inexpensive, and environmentally benign. In this work, an organic–inorganic hybrid based on xanthan gum, carboxymethyl cellulose, and silica (XG/CMC/silica) was produced by a sol–gel approach using 3-aminopropyltriethoxysilane (APTES) as the silica-forming and amine-bearing precursor. The hybrid was examined by FTIR, XRD, SEM-EDX, and TGA. FTIR spectra verified the coexistence of polysaccharide bands and Si–O–Si vibrations, whereas XRD showed that the silica phase lowered the ordered domains of the XG/CMC blend and generated a largely amorphous hybrid network. SEM-EDX images revealed an open, rough, and porous architecture in which silicon was distributed throughout the matrix. TGA further showed enhanced thermal resistance, with a residual mass of about 49% at 600 °C for the silica-containing material. The adsorbent was tested for the uptake of methyl orange (MO) and methylene blue (MB) as representative anionic and cationic dyes. Dye removal depended strongly on pH; MB uptake was highest at pH 6, while MO removal was favored at pH 3, reflecting the contribution of deprotonated carboxylate/silanol sites and protonated amine groups, respectively. The kinetic profiles agreed better with the pseudo-first-order equation. The Langmuir provided the best representation of the data of equilibrium, giving maximum capacities of 116 and 107 mg g^−1^ for MB and MO. Thermodynamic analysis confirmed that the adsorption process was spontaneous, with Δ*G* values ranging from −1.5 to −2.1 kJ mol^−1^ for MB and −0.7 to −1.3 kJ mol^−1^ for MO, and endothermic, with Δ*H* values of 7.13 kJ mol^−1^ (MB) and 7.86 kJ mol^−1^ (MO). Furthermore, the hybrid retained useful adsorption capacity after five adsorption–desorption cycles, demonstrating good reusability. The XG/CMC/silica hybrid represents a promising, reusable, bio-based adsorbent for the efficient removal of both cationic and anionic dyes from aqueous media.

## Abstract 2

The XG/CMC/silica hybrid adsorbent was successfully prepared by sol–gel method using APTES. The hybrid showed high adsorption capacities of 116 mg g^−1^ for MB and 107 mg g^−1^ for MO. The material demonstrated excellent reusability after 5 cycles.

## Graphical abstract

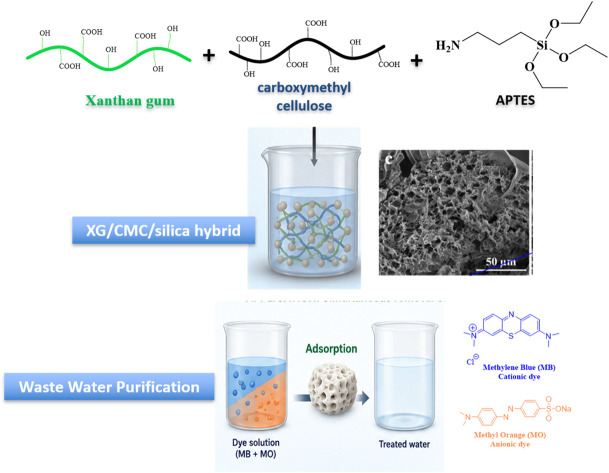

## 1 Introduction

Wastewaters discharged from dye-consuming industries are a persistent environmental problem because many synthetic colorants are chemically stable, intensely colored, and resistant to biodegradation. Large quantities of commercial dyes are produced every year, and a considerable fraction may enter surface waters when treatment is insufficient.^1,2^ These compounds are used in textile, pharmaceutical, printing, cosmetic, and related sectors; once released, they can block light penetration, disturb photosynthetic activity, and introduce toxic, or poisonous risks to aquatic organisms and humans.^3,4^ Therefore, practical treatment technologies capable of removing dissolved dyes are urgently needed.^5^ Among the available biological, chemical, and physical processes, adsorption remains attractive because it is simple to operate, can be applied at relatively low cost, and often provides high removal efficiency under mild conditions.^6,7^

In this context, biopolymers such as polysaccharides have emerged as promising adsorbents due to their renewability, biodegradability, and rich surface functionalities capable of interacting with various dye molecules.^8^ Xanthan gum (XG) is a microbial exopolysaccharide widely recognized for its biodegradability, non-toxicity, and excellent physicochemical properties, making it valuable in various applications such as wastewater treatment, protein extraction, drug delivery, tissue engineering, and food packaging.^9,10^ The structure possesses a negative charge due to a polysaccharide backbone made of cellulose, with trisaccharide side chains that include glucuronic acid and pyruvic acid.^11^ Its high viscosity, water solubility, and abundance of hydroxyl and carboxyl functional groups allow effective interaction with pollutants, particularly organic dyes.^12^ Despite these advantages, XG suffers from certain drawbacks, including poor mechanical strength, limited thermal stability, and sensitivity to hydration, which can restrict its practical use under harsh environmental conditions. Similarly, a water-soluble cellulose derivative, carboxymethyl cellulose (CMC), provides a high density of negatively charged carboxyl groups, offering strong interactions with cationic dyes.^13,14^ However, its mechanical robustness and chemical stability are also limited when used alone in aqueous environments. To overcome the integral limitations of biopolymers such as low mechanical strength and poor thermal stability the incorporation of inorganic components like silica into biopolymer matrices has been widely explored. Silica is a chemically inert, thermally stable, and highly porous material characterized by a large surface area and tunable surface chemistry, making it particularly suitable for adsorption applications.^15^ When integrated into polymeric networks, especially *via* sol–gel processes, silica forms an interpenetrating hybrid structure that enhances the mechanical strength, thermal resistance, and structural integrity of the resulting biocomposites. Moreover, chemical functionalization of silica introduces additional active sites on the surface, further increasing the adsorption capacity toward various pollutants, including dyes.^16^

The sol–gel method introduces a versatile and mild synthetic route for the producing of organic–inorganic hybrid materials.^17^ It involves the hydrolysis and condensation of metal alkoxides, most commonly tetraethyl orthosilicate (TEOS) to form a silica network within a liquid medium, which gradually transitions into a gel.^18^ When applied to biopolymer-based systems, this approach enables the uniform incorporation of silica into natural polymer matrices, yielding porous, mechanically robust hybrid structures. These materials retain the functional groups and biocompatibility of the biopolymers while benefiting from the added thermal stability and adsorptive efficiency provided by the silica network. Sol–gel produced xanthan gum/silica and CMC/silica composites have been shown in earlier research to be successful in removing heavy metals and dyes from aqueous media, underscoring their promise as multipurpose materials for environmental remediation. For example, Thakur *et al.* synthesized a xanthan gum/silica (XG/SiO_2_) hybrid by the sol–gel method. The resulting material exhibited well-organized macro- and mesoporous structures, a high surface area, excellent chemical and mechanical stability, and noticeable adsorption capacities for methylene blue (MB) and bromophenol blue (BB) dyes.^19^ A comparable nanoadsorbent with significant antibacterial activity was established in another work by ultrasonicating xanthan gum and SiO_2_ nanoparticles, then crosslinking polymerization with vinyl imidazole. The resulting XG-g-PVI/SiO_2_ composite demonstrated high efficiency and rapid adsorption performance for the dye removal from aqueous media, highlighting its potential in wastewater treatment applications.^20^ Likewise, CMC/silica composites prepared through the sol–gel process have shown excellent adsorption capabilities due to the synergistic interaction between the carboxyl and hydroxyl groups of CMC and the silanol groups of silica. These hybrids not only offer a high density of active binding sites but also exhibit enhanced thermal and mechanical stability. Furthermore, the incorporation of complementary functional components into hybrid adsorbents has been shown to improve structural stability while creating additional adsorption-active sites, resulting in superior adsorption performance through synergistic interactions between the individual constituents.^21^ Several reports have confirmed their ability to effectively remove pollutants such as lead(ii), cadmium(ii), and various organic dyes from water, making them attractive candidates for use in sustainable water purification technologies.^22,23^ Furthermore, the incorporation of porous carbonaceous materials has been shown to further improve adsorption performance by providing abundant active sites, facilitating mass transfer, and promoting interactions between pollutants and the adsorbent surface.^24,25^ To the best of our knowledge, the integration of xanthan gum, carboxymethyl cellulose (CMC), and silica into a single hybrid material for the simultaneous removal of both cationic and anionic dyes has not been previously reported, representing a novel and promising strategy for wastewater treatment. The combination of natural biopolymers with functionalized silica precursors provides an effective and low-cost route to fabricate hybrid materials with enhanced adsorption capacity and decontamination efficiency.

The present study focuses on the synthesis and characterization of a XG/CMC/silica hybrid incorporating both carboxylic and amino functional groups. XG and CMC were solubilized in water and subsequently mixed with a silica precursor, followed by synthesis *via* the sol–gel process. The resulting hybrid was investigated as a novel bioadsorbent to remove the organic dyes from aqueous media. Additionally, adsorption modeling was performed to determine the maximum adsorption capacity, evaluate the mechanism of adsorption, and assess the thermodynamic parameters governing the process.

## 2 Experimental section

### 2.1. Materials and sample preparation

**Fig. 1:**
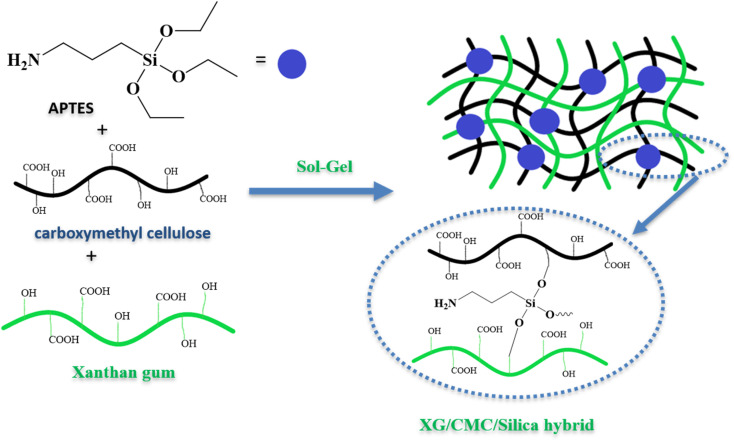
Schematic procedure for preparing the XG/CMC/silica hybrid material.

Xanthan gum (from Xanthomonas campestris), carboxymethyl cellulose (CMC), methylene blue (MB) and methyl orange (MO) were bought from Sigma-Aldrich (St. Louis, MO, USA). The XG/CMC/silica porous hybrid was prepared according to the procedure illustrated in Fig. 1. First, native xanthan gum was deacetylated following the method reported by Černík and Vinod Vellora^26^ with slight modification. A 1.0% (w/v) dispersion of native xanthan gum in deionized water was kept at constant temperature for 12 h to allow separation of undissolved matter. The mixture was filtered to obtain a clear solution, and deacetylation was performed by adding 1.0 M NaOH under gentle stirring at room temperature. The pH was then adjusted to 7.0 using 1.0 M HCl, and the neutralized solution was freeze-dried to obtain deacetylated xanthan gum powder. The deacetylation treatment was carried out to expose additional hydroxyl groups by removing acetyl substituents from the xanthan gum structure. This modification enhances the interaction of xanthan gum with the silica network formed during the sol–gel process and increases the availability of adsorption-active sites, thereby improving the structural integrity and adsorption performance of the resulting XG/CMC/silica hybrid. To prepare the porous hybrid, 50 mL of a 10.0% (w/v) deacetylated XG solution and 50 mL of a 2.0% (w/v) CMC solution were mixed (1 : 1, w/w) and mechanically stirred at 5000 rpm for 10 min at room temperature. Subsequently, 40 mmol of 3-aminopropyltriethoxysilane (APTES, 9.4 mL) was added to the mixture, followed by stirring at 1000 rpm for 30 min. Thereafter, 1 mL of 0.1 M HCl was added to initiate the sol–gel reaction, and the reaction mixture was continuously stirred at 300 rpm for 48 h. The resulting whitish gel was cast into molds and freeze-dried at −80 °C to obtain the porous XG/CMC/silica hybrid. During the sol–gel process, APTES undergoes hydrolysis to form silanol groups (Si–OH), followed by condensation to generate siloxane bonds (Si–O–Si). These reactions create a silica-rich network within the XG/CMC matrix and introduce amino groups that can participate in the adsorption of anionic dyes. The hydroxyl and carboxyl groups of XG and CMC can further interact with silanol/siloxane groups through hydrogen bonding and possible condensation reactions, contributing to the structural integration of the hybrid matrix.

### 2.2. Characterization methods

Fourier Transform Infrared (FTIR) spectroscopy was performed of synthesized materials and study possible intermolecular interaction between XG/CMC polymer matrix and embedded silica phase. Spectra were recorded in the range 4000–400 cm of wavenumber using Bruker FTIR spectrometer and KBr-pellet samples. Crystal structure and phase organization of synthesized materials were investigated using X-ray diffraction (XRD) by Rigaku diffractometer with Cu K radiation (*λ* = 1.5406 Å). Measurements were carried out at 40 kV and 30 mA in the 2 of 5–80 with a scan rate of 2 min, to investigate possible differences in crystallinity after introduction of silica in XG/CMC framework. Thermal behavior of the synthesized materials and their thermal degradation were studied by thermogravimetric analysis (TGA) carried out on a TA Instruments TGA Q500 analyzer where each sample (∼5 mg, dried) was heated in nitrogen from 25 to 600  C at a heating rate of 10  C min. Finally, surface morphology and microstructure were observed by scanning electron microscopy (SEM) using a JEOL JSM-IT500 instrument. Samples were coated with gold for 30 s. To avoid electrical charging under scanning conditions. In addition, energy-dispersive X-ray spectroscopy and elemental mapping were employed to identify the elemental composition of the hybrid and to examine the distribution of the main elements within the sample structure.

### 2.3. Adsorption studies of cationic and anionic dyes

The removal efficiency of MB and MO dye onto XG/CMC/silica hybrid adsorbent were investigated through batch adsorption experiments. In a general case, 50 mg of adsorbent was added to 100 mL of dye solution at certain initial concentration and varied working conditions such as solution pH, adsorbent dosage, contact time, initial dye concentration and temperature were studied. Except for indicated otherwise, all experiments were carried out with continuous stirring at room temperature. After adsorption, the suspensions were separated by centrifugation, and 3 mL aliquots of the supernatant were collected for analysis. The residual concentrations of MB and MO were determined using a UV-Vis spectrophotometer at *λ*_max_ = 664 nm for MB and *λ*_max_ = 464 nm for MO. The equilibrium adsorption capacity was calculated using eqn (1):1
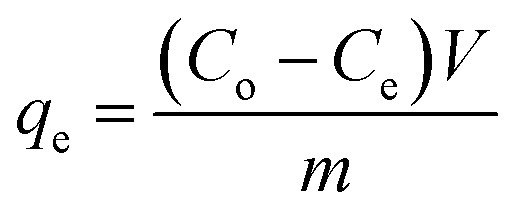
where *q*_e_ (mg g^−1^) denotes the amount of dye adsorbed per gram of adsorbent at equilibrium, *C*_0_ and *C*_e_ (mg L^−1^) represent the initial and equilibrium dye concentrations in the solution, respectively, *V* (L) is the volume of the dye solution, and *m* (g) is the mass of the XG/CMC/silica hybrid adsorbent. All measurements were carried out in triplicate, and the results are presented as the mean accompanied by the standard deviation. Statistical evaluations were performed using OriginPro, and differences were deemed significant when *p* < 0.05.

## 3 Results and discussion

### 3.1. Characterization of the samples

#### 3.1.1. FTIR characterization

**Fig. 2:**
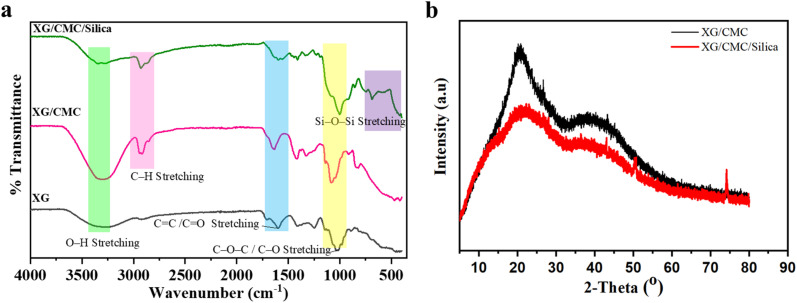
(a) FTIR spectrum of XG, XG/CMC, and XG/CMC/silica hybrid (b) XRD of XG/CMC, and XG/CMC/silica hybrid.

The FTIR spectra of XG, XG/CMC, and XG/CMC/silica hybrid are presented in Fig. 2. These spectra provide insight into the chemical structure and functional group interactions involved in the formation of the hybrid materials. A broad absorption band observed in the range 3300–3400 cm^−1^ corresponds to O–H stretching vibrations, which is characteristic of hydroxyl groups involved in extensive hydrogen bonding, typical in polysaccharides. The presence of C–H stretching vibrations around 2920 and 2850 cm^−1^ indicates aliphatic –CH_2_ and –CH_3_ groups within the polymer backbone. A distinct peak near 1600 cm^−1^ is due to C=O stretching from carboxylic acid groups in CMC and possibly C=C stretching associated with the glucuronic acid moieties in xanthan gum. In the fingerprint region, intense bands between 1200 and 1000 cm^−1^ are attributed to C–O–C and C–O stretching, which confirm the presence of ether and alcohol functionalities inherent to the polysaccharide structure.^27,28^ Significantly, the spectrum of the XG/CMC/silica composite displays additional bands at ∼1100 cm^−1^, 800 cm^−1^, and 470 cm^−1^, which are attributed to the asymmetric stretching and bending modes of Si–O–Si bonds, respectively.^29^ The emergence and intensification of these bands confirm the successful incorporation of silica into the hybrid matrix. Furthermore, shifts and changes in intensity of the main absorption bands suggest strong intermolecular interactions, between the organic components and the silica phase such as hydrogen bonding.

#### 3.1.2. XRD characterization

The XRD patterns of XG/CMC and the XG/CMC/silica hybrid are presented in Fig. 2b. The XG/CMC sample displayed a broad diffraction band centered around 2*θ* = 20 and 22°, which is characteristic of the semi-crystalline nature of cellulose-based polysaccharide materials.^30^ This broad peak indicates the presence of partially ordered regions originating mainly from CMC chains, together with the amorphous contribution of xanthan gum. A second broad feature was also observed around 2*θ* = 38–42°, suggesting limited molecular ordering within the blended biopolymer matrix. After the incorporation of silica, the XG/CMC/silica hybrid exhibited a clear decrease in diffraction intensity and broader diffraction features compared with the XG/CMC sample. This reduction in peak intensity indicates a decrease in the degree of crystallinity of the polymeric matrix after sol–gel silica formation. The broad halo observed in the range of approximately 20–30° is consistent with the presence of amorphous silica. The absence of sharp crystalline silica peaks suggests that the silica phase was mainly formed in an amorphous state and was well dispersed within the XG/CMC network. The decrease in crystallinity may be attributed to the interaction between silanol/siloxane groups and the hydroxyl and carboxyl groups of XG and CMC, which disrupts the regular packing of the polysaccharide chains. These results are in agreement with the FTIR observations, where the appearance and intensification of Si–O–Si related bands confirmed the successful incorporation of silica. The XRD results demonstrate that the sol–gel process produced an amorphous organic–inorganic hybrid structure rather than a separate crystalline silica phase.

### 3.2. Thermal analysis

**Fig. 3:**
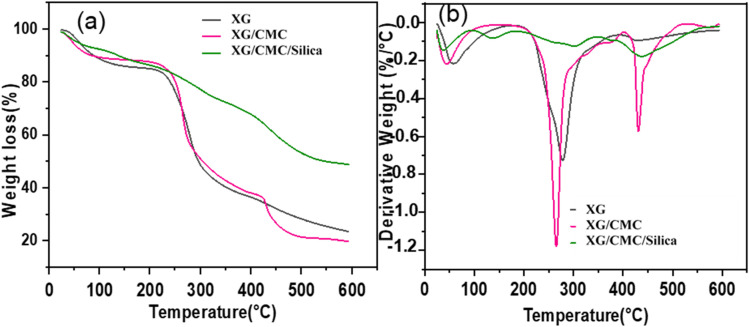
(a) TGA and (b) DTG curves of XG/CMC and XG/CMC/silica hybrid.

The thermal stability of XG, XG/CMC, and the XG/CMC/silica hybrid was evaluated by TGA over the range of 25–600 °C, as shown in Fig. 3a. Native XG showed an initial mass loss of 13.3% below 130 °C, which is attributed mainly to moisture evaporation. The main degradation stage began at approximately 216 °C and continued to about 330 °C, corresponding to polysaccharide-chain decomposition. A further gradual mass loss occurred from 330 to 600 °C due to continued carbonization and degradation of the organic matrix. The XG/CMC sample showed a degradation profile similar to that of XG, reflecting the comparable polysaccharide nature of the blend. The thermal degradation of CMC is commonly associated with dehydration, formation of polyene-type intermediates, and subsequent decomposition into carbonaceous residues and volatile products.^31^ In contrast, the XG/CMC/silica hybrid degraded more gradually and retained approximately 49% residual mass at 600 °C. This higher residue confirms the successful incorporation of the inorganic silica phase and indicates improved thermal stability of the hybrid network.

### 3.3. SEM analysis

**Fig. 4:**
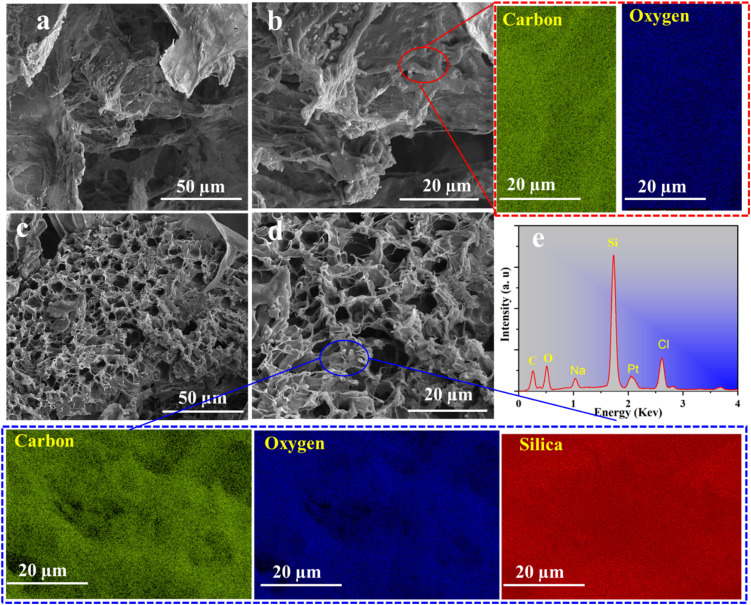
SEM images of XG/CMC (a and b) and XG/CMC/silica hybrid (c and d), elemental mapping of XG/CMC and XG/CMC/silica hybrid and EDX spectra of and XG/CMC/silica hybrid (e).

The surface morphology and elemental composition of the XG/CMC and XG/CMC/silica hybrid were investigated using SEM coupled with EDX analysis, as shown in Fig. 4. The SEM images of the XG/CMC sample (Fig. 4a and b) reveal a relatively compact and dense morphology with limited visible pores. This morphology is likely related to the strong intermolecular hydrogen bonding between xanthan gum and CMC chains, which promotes extensive chain aggregation and reduces the development of open porous networks. This compact surface structure is expected to hinder the diffusion and accessibility of dye molecules to internal adsorption sites, thereby limiting the overall adsorption efficiency.^32,33^ In contrast, the XG/CMC/silica hybrid (Fig. 4c and d) exhibits a markedly different morphology, characterized by a rough, interconnected, and highly porous network. The formation of this open structure can be attributed to the sol–gel reaction of APTES, where hydrolyzed silanol groups undergo condensation to form a silica-based network within the XG/CMC matrix. The incorporation of silica appears to disturb the compact arrangement of the biopolymer chains, generating a more irregular and porous architecture. This morphology is advantageous for adsorption because it can provide more accessible surface area, facilitate dye diffusion into the hybrid matrix, and expose a larger number of active functional groups, such as hydroxyl, carboxyl, amino, and silanol groups.^34^ Therefore, the porous structure observed in the XG/CMC/silica hybrid is expected to contribute significantly to the enhanced adsorption of both anionic MO and cationic MB dyes. The EDX spectrum of the XG/CMC/silica hybrid confirms the presence of carbon, oxygen, and silicon. Carbon and oxygen originate mainly from the polysaccharide backbone of xanthan gum and CMC, while the strong silicon signal confirms the successful incorporation of the silica phase into the hybrid structure. The detection of silicon provides direct evidence that APTES participated in the formation of the organic–inorganic network during the sol–gel process.^35^ In addition, the elemental mapping images show a relatively uniform distribution of carbon, oxygen, and silicon throughout the hybrid matrix. This homogeneous distribution indicates that the silica phase was not simply deposited as large, separated particles on the surface, but was well integrated within the XG/CMC network. The uniform dispersion of silicon is particularly important because it suggests the successful formation of a continuous organic–inorganic hybrid structure. Such integration can improve the structural stability of the adsorbent and increase the availability of silanol and amino-containing sites for dye binding. The presence of oxygen-rich domains also supports the abundance of hydroxyl, carboxylate, and silanol groups, which are important for hydrogen bonding and electrostatic interactions with dye molecules. The SEM and EDX results strongly support the successful fabrication of a porous XG/CMC/silica hybrid. These findings are consistent with the FTIR and XRD results, which confirmed the formation of Si–O–Si linkages and the development of a more amorphous hybrid structure after silica incorporation.

### 3.4. Adsorption of MB and MO dyes

XG/CMC/silica hybrid was explored as an efficient bioadsorbent for both MO and MB dyes adsorption which were selected as examples for anionic and cationic dyes, and the adsorption method was examined. Adsorption capacities of XG/CMC/silica hybrid was observed under various pHs, contact times, initial dye concentrations and temperatures.

#### 3.4.1. Effect of pH

**Fig. 5:**
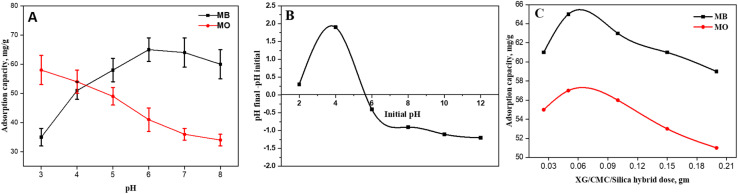
Effect of pH and adsorbent dose of XG/CMC/silica hybrid on MB and MO adsorption (A and C). Point zero charge of XG/CMC/silica hybrid (B).

The pH of the aqueous solution plays a decisive role in dye adsorption by modulating both the surface charge of the adsorbent and the ionization state of the dye molecules. As shown in Fig. 5A, the adsorption performance of the XG/CMC/silica hybrid varied markedly with pH. For MB, the adsorption capacity increased as the pH was raised, reaching the highest value of 65 mg g^−1^ at pH 6. This behavior can be explained by the progressive deprotonation of hydroxyl, carboxyl, and silanol groups, which generates more negatively charged sites on the hybrid surface. These negatively charged sites promote electrostatic attraction with the cationic MB molecules, resulting in improved adsorption under near-neutral conditions.^16^ In contrast, MO adsorption was favored under acidic conditions, with the maximum adsorption capacity observed at pH 3. At low pH, the amino groups introduced through APTES become protonated to form –NH_3_^+^ groups, creating positively charged adsorption sites. These sites enhance the electrostatic attraction between the hybrid surface and the anionic MO molecules. As the pH increases, deprotonation of the amino groups reduces the positive surface charge, weakening the interaction with MO and consequently decreasing its adsorption capacity. This pH-dependent adsorption behavior confirms that the XG/CMC/silica hybrid contains dual functional domains: carboxylate-rich sites that favor cationic dye adsorption and protonated amino sites that favor anionic dye adsorption. The material also remained stable and insoluble over the investigated pH range, indicating its suitability for the adsorption of both cationic and anionic dyes from aqueous media. The point of zero charge (pH_pzc_) further supports the pH-dependent adsorption mechanism. As shown in Fig. 5B, the pH_pzc_ of the XG/CMC/silica hybrid was 6.5, indicating that the surface charge changes from positive to negative around this value. Below the pH_pzc_, protonation of amino groups gives the adsorbent a positively charged surface, which is favorable for MO adsorption. Above the pH_pzc_, the surface becomes predominantly negative due to the deprotonation of carboxyl, hydroxyl, and silanol groups, thereby enhancing the uptake of MB. Therefore, the adsorption behavior of MB and MO is mainly governed by the balance between surface protonation/deprotonation and electrostatic interactions between the dyes and the active functional groups of the hybrid. The effect of adsorbent dosage on MB and MO adsorption is shown in Fig. 5C. Increasing the amount of XG/CMC/silica hybrid initially enhanced the adsorption capacity of both dyes, with the optimum dose observed at 0.05 g. This improvement is associated with the increased number of available adsorption sites, including hydroxyl, carboxylate, amino, and silanol groups, which can interact with dye molecules through electrostatic attraction and hydrogen bonding. The higher adsorption capacity observed for MB compared with MO suggests stronger affinity between the cationic MB molecules and the negatively charged groups present in the XG/CMC/silica network. However, further increasing the adsorbent dose led to a reduction in adsorption capacity per unit mass. This decline may be attributed to particle aggregation, overlapping of active sites, and reduced accessibility of the internal porous structure. At higher solid concentrations, the effective surface area available for dye binding can decrease, and mass-transfer limitations may become more pronounced. These results indicate that 0.05 g is the most suitable adsorbent dosage under the conditions studied.

**Fig. 6:**
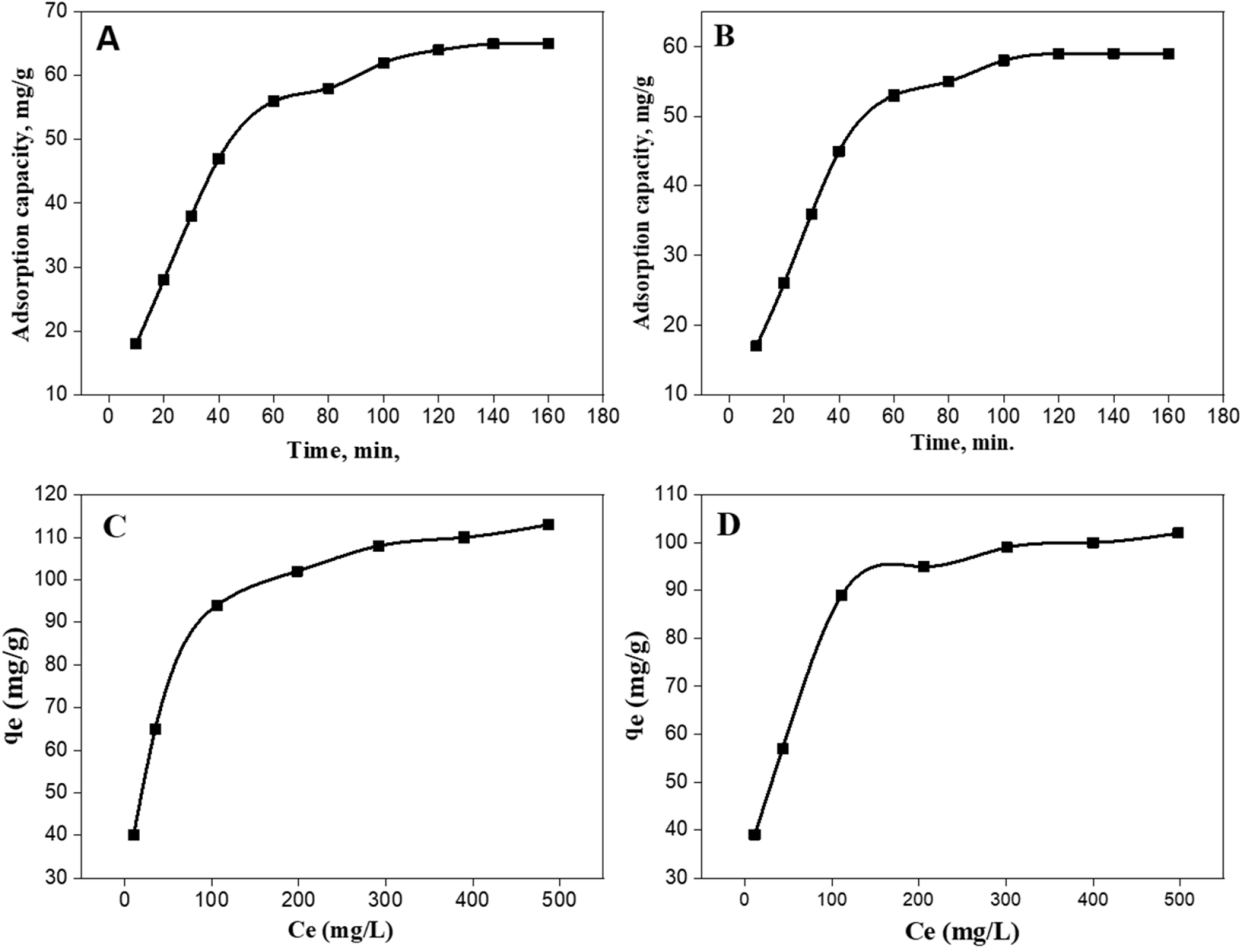
Contact time-dependent adsorption of MB (A) and MO (B) onto the XG/CMC/silica hybrid, and equilibrium adsorption isotherms (*q*_e_*versus C*_e_) for MB (C) and MO (D).

The influence of contact time was investigated to assess the adsorption kinetics and equilibrium behavior of the XG/CMC/silica hybrid toward MB and MO dyes. As illustrated in Fig. 6A, the adsorption capacity increased rapidly during the initial stage of the process, indicating the abundance of readily available active sites on the hybrid surface. This fast uptake can be attributed to the easy accessibility of hydroxyl, amino, carboxylate, and silanol groups, as well as the porous structure of the hybrid, which facilitates dye diffusion and surface interaction. After the initial rapid adsorption stage, the uptake rate gradually decreased and approached equilibrium after approximately 60 min. This slower stage is likely due to the progressive occupation of active sites and the reduction in the concentration gradient between the adsorbent surface and dye solution. At equilibrium, the adsorption capacities reached 65 mg g^−1^ for MB and 58 mg g^−1^ for MO. The higher adsorption capacity of MB may be associated with the strong electrostatic attraction between the cationic MB molecules and the negatively charged carboxylate-rich sites originating from XG and CMC. In contrast, MO adsorption is mainly promoted by the protonated amino groups introduced through the silica precursor, which provide positively charged sites for interaction with the anionic dye molecules. In addition to electrostatic attraction, hydrogen bonding between dye molecules and functional groups such as –OH, –COOH/–COO^−^, –NH_2_/–NH_3_^+^, and Si–OH may also contribute to the adsorption process.^36^

The adsorption kinetics of MB and MO onto the XG/CMC/silica hybrid were evaluated using the pseudo-first order, pseudo-second-order and the Elovich models, as presented in eqn (2)–(4).

2 *q*_*t*_ = *q*_e_(1 − e^(−*k*_1_*t*)^)

3
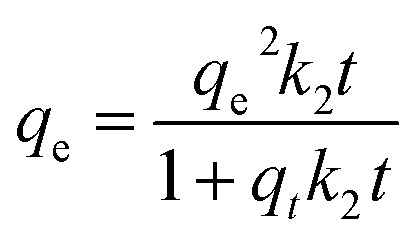
4
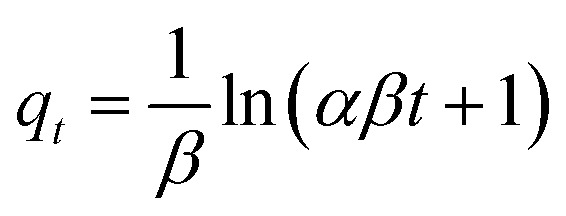
where *q*_e_ and *q*_*t*_ (mg g^−1^) represent the adsorption capacities at equilibrium and at time *t* (min), respectively Additionally, *k*_1_ and *k*_2_ describe corresponding rate constants. The data fitted to three different models, and the kinetic parameters are listed in Table 1. Comparison of the correlation coefficients (*R*^2^) indicates that the pseudo-first-order model provides the best description of the adsorption kinetics for both dyes, with *R*^2^ values of 0.98 for MB and MO. These results suggest that the adsorption rate is mainly governed by the availability of adsorption sites during the initial stage of the adsorption process. In comparison, the pseudo-second-order model predicted a higher adsorption capacity but exhibited a lower coefficient of determination (*R*^2^ = 0.94 and 0.96 for MB and MO, respectivelly), indicating a less satisfactory fit. To further investigate the adsorption mechanism, the nonlinear Elovich model was also applied. The relatively high values of *α* indicate a rapid initial adsorption rate due to the abundance of available active sites on the XG/CMC/silica surface. The low *β* values suggest that the adsorption rate gradually decreases as surface coverage increases. However, the comparatively lower *R*^2^ values demonstrate that the Elovich model does not describe the adsorption kinetics as effectively as the pseudo-first-order model. This finding implies that although surface heterogeneity and chemisorption may contribute to the adsorption process, they are unlikely to be the sole rate-controlling mechanisms. The kinetic analysis demonstrates that the adsorption of both MB and MO onto the XG/CMC/silica hybrid is best represented by the pseudo-first-order model, whereas the pseudo-second-order and Elovich models provide complementary information regarding the adsorption process.

**Table 1:** Kinetic parameters of MB and MO adsorption using XG/CMC/silica hybrid

Models	Parameters	MB	MO
Pseudo first order	*q*_e,cal_ (mg g^−1^)	91.5	59.7
	*k*_1_ (min^−1^)	0.04	0.04
	*R* ^2^	0.98	0.98
Pseudo second-order	*q*_e,cal_ (mg g^−1^)	107.6	70.3
	*k*_2_ (g mg^−1^ min^−1^)	4.4 × 10^−4^	6.4 × 10^−4^
	*R* ^2^	0.94	0.96
Elovich	*β* (g mg^−1^)	0.04	0.07
	*α* (mg (g min)^−1^)	10.2	6.2
	*R* ^2^	0.87	0.91

#### 3.4.2. Effect of dye concentration and equilibrium isotherm

Fig. 6 shows how the adsorption capacity changed with respect to the equilibrium dye concentration. The adsorption capacity increased along with the equilibrium dye concentration; the reason lies in the increase of the concentration gradient between solution bulk and solid surface. The greater concentration gradient leads to the enhancement of dye moving from liquid phase to the active sites present in the hybrid material. At the equilibrium dye concentration to 390 and 400 mg L^−1^, the adsorption capacities achieved 110 and 100 mg g^−1^ for MB and MO, respectively. MB uptake ability is higher because the electrostatics interaction between cationic dye and negative charged carboxylate groups within hybrid matrix was stronger than MO. To describe the equilibrium characteristic of adsorption process, the experiment results were fitted by Langmuir and Freundlich isotherms. The considered results were calculated by Langmuir model which is presented by eqn (5).5
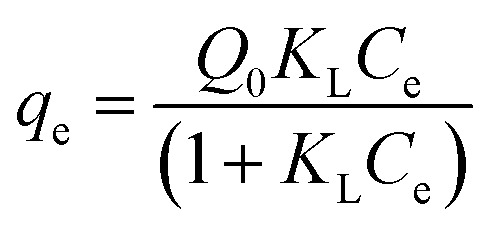
In the Langmuir equation, *K*_L_ reflects the solute absorptivity (L g^−1^) and *Q*_0_ is the Langmuir adsorption capacity (mg g^−1^). In addition, *C*_e_ represents the equilibrium dye concentration in solution (mg L^−1^), (*q*_e_) denotes the *t* equilibrium adsorption capacity. To comprehensively evaluate the fitting accuracy and validate the suitability of the applied isotherm models, multiple error structure functions the coefficient of determination *R*^2^, root mean square error (RMSE), normalized standard deviation Δ*Q* (%), and the chi-squared statistic *χ*^2^ were determined. As compiled in Table 2, the Langmuir model described the adsorption behavior of both dyes significantly more effectively compared to the Freundlich model. This superiority is clearly evidenced by the higher correlation coefficients *R*^2^ = 0.9846 for MB and 0.9513 for MO, minimized RMSE values 2.88 and 3.14 for MB and MO, respectively, and noticeably low chi-squared metrics *χ*^2^ = 1.24 for MB and 1.56 for MO presented in Table 2. Furthermore, the normalized standard deviations Δ*Q* (%) for the Langmuir model remained well within highly acceptable thresholds 6.36 for MB and 7.12 for MO. These minimized residual errors securely confirm that MB and MO adsorption mainly occurred through a uniform monolayer coverage on the homogeneous, available active sites of the XG/CMC/silica hybrid matrix.

**Table 2:** The adsorption parameters of organic dyes by xanthan gum/CMC/silica hybrid according to different equilibrium patterns

Models	Parameters	MB	MO
Langmuir	*Q*_o_ (mg g^−1^)	116.135	107
	*K*_L_ (L mg^−1^)	0.0422	0.0374
	*R* ^2^	0.9846	0.9513
	RMSE	2.88	3.14
	Δ*Q* %	6.36	7.12
	*χ* ^2^	1.24	1.56
Freundlich	*K*_F_ (mg^1−*b*_F_^ L^*b*_F_^ g^−1^)	28.9282	26.2983
	*b*_F_	4.3728	4.3776
	*R* ^2^	0.932	0.899
	RMSE	6.04	6.58
	Δ*Q* %	10.42	11.85
	*χ* ^2^	3.53	4.12

The adsorption capacity for Langmuir was calculated to be 116 mg g^−1^ for MB and 107 mg g^−1^ for MO. These relatively high values confirm the important contribution of the hybrid structure and its multiple functional groups to dye uptake. The carboxylate- and hydroxyl-rich domains of XG and CMC provide favorable binding sites for cationic MB, while the amino and silanol groups introduced through the silica precursor contribute to the adsorption of anionic MO. Therefore, the adsorption performance can be attributed to the combined effects of electrostatic attraction, hydrogen bonding, and the porous organic–inorganic network.

The equilibrium data were also analyzed using the Freundlich model, expressed in eqn (6).

6 *q*_e_ = *K*_F_*C*_e_^*b*_F_^

In this model, *K*_F_ reflects the adsorbent capacity and *b*_F_ is the heterogeneity factor (unitless) ranging from 0 to 1. The Freundlich model yielded correlation coefficients of 0.93 for MB and 0.89 for MO, which are lower than those obtained from the Langmuir model. This indicates that the Freundlich isotherm provided a poorer fit for describing dye adsorption on the XG/CMC/silica hybrid. The calculated *K*_F_ values were 28.9 and 26,2 for MB and MO, respectively, while the corresponding *b*_F_ values were 4.37 and 4.38. However, the better agreement with the Langmuir model indicates that adsorption mainly proceeded through uniform monolayer adsorption rather than multilayer adsorption on highly heterogeneous surfaces.

#### 3.4.3. Temperature and thermodynamic study

The thermodynamic behavior of MB and MO adsorption onto the XG/CMC/silica hybrid was investigated by conducting adsorption experiments at different temperatures of (298, 308, and 318 K).

Key thermodynamic parameters, namely the standard enthalpy change (Δ*H*°), standard entropy change (Δ*S*°), and standard Gibbs free energy change (Δ*G*°), were determined to evaluate the spontaneity and thermal nature of the adsorption process. The Gibbs free energy change was calculated using eqn (7):

7 Δ*G*° = −*RT* ln *K*_c_

8
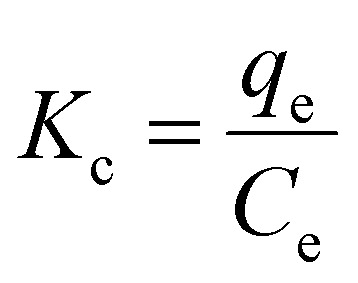
*K*_c_ is the distribution coefficient, *R* is the universal gas constant (8.314 J mol^−1^ K^−1^), and *T* is the absolute temperature.9
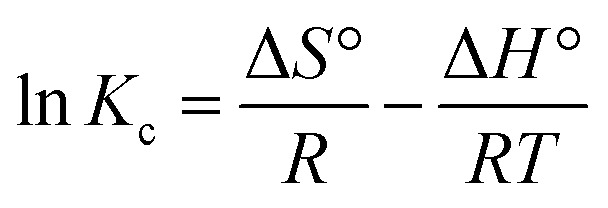
where the quantities of Δ*H*° and Δ*S*° are calculated from the slope and intercept of the linear draw of ln *K*_c_*vs.*1/*T*.

**Fig. 7:**
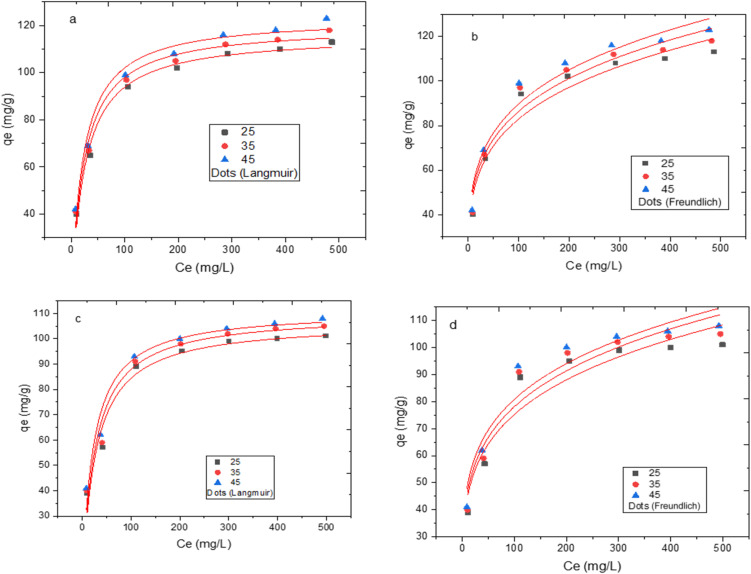
Adsorption isotherms for MB and MO on the XG/CMC/silica hybrid at different temperatures. (a and c) Present the experimental results with the corresponding the non-linear fitting of the Langmuir model, while (b and d) illustrate the fitting of the Freundlich model.

The effect of temperature on the adsorption performance was further investigated, and the equilibrium data were fitted using the non-linear Langmuir and Freundlich isotherm models, as shown in Fig. 7. Both dyes' adsorption capacities enhanced with the increase in temperature from 25 °C to 45 °C. That is, for the initial dye concentration of 600 mg L, the uptake of MB was 113 mg g^−1^ when temperature is 25 °C and reached 123 mg g^−1^ at 45 °C. However, the adsorption of MO was 101 mg g^−1^ and enhanced to 108 mg g^−1^ as temperature was 25 °C and 45 °C respectively. This indicates that adsorption is endothermic and high temperatures are favorable for adsorption. These results could be due to enhanced molecular diffusion and mobility of dyes and increase in available adsorption sites in porous structure of hybrid at higher temperatures.

**Fig. 8:**
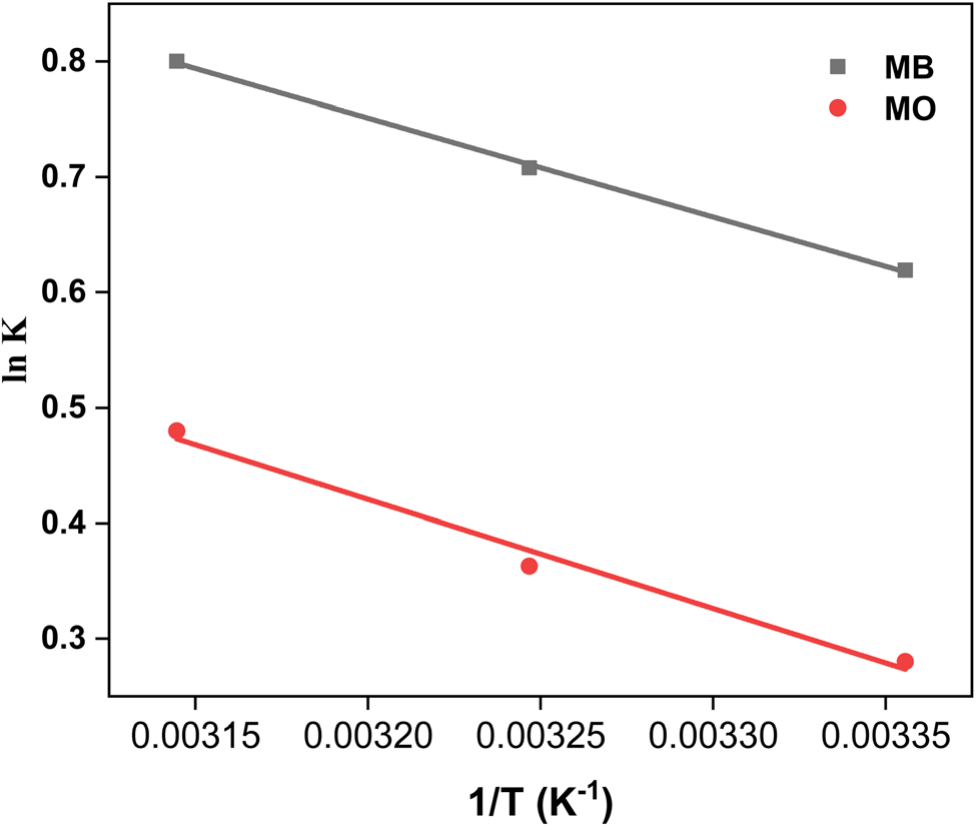
Effect of temperature on the adsorption process, including the Van't Hoff plot of ln(*K*) *versus* 1/*T*.

The thermodynamic parameters were calculated from the Van't Hoff plot presented in Fig. 8 and are summarized in Table 3. This suggests that increased thermal energy promotes dye diffusion and exposure of more binding sites. Negative Δ*G*° values at all temperatures indicate that the process is spontaneous and favorable, while positive Δ*S*° values reflect greater disorder at the solid–liquid interface. This increase in entropy may result from the release of water molecules that initially surround the dye molecules in solution. During adsorption, these hydration layers are partially disrupted as dye molecules interact with the functional groups of the XG/CMC/silica hybrid, leading to greater disorder in the system.

**Table 3:** Thermodynamic parameters for the adsorption of MB and MO adsorption onto XG/CMC/silica hybrid

Dye	Δ*H*° (kJ mol^−1^)	Δ*S*° (kJ mol^−1^ K^−1^)	Δ*G*° (kJ mol^−1^)		
			298 K	308 K	318 K
MB	7.13	29	−1.5	−1.8	−2.1
MO	7.86	20.3	−0.7	−0.9	−1.3

Furthermore, the hybrid surface contains different active groups, including carboxylate, hydroxyl, amino, and silanol groups. These groups facilitate dye binding through electrostatic attractions and hydrogen bonding. These multiple interaction sites may allow dye molecules to adopt different adsorption orientations on the adsorbent surface, further contributing to the increase in interfacial disorder. The thermodynamic results confirm that MB and MO adsorption onto the XG/CMC/silica hybrid is spontaneous, endothermic, and favored by increased temperature, supporting the potential of this material as an efficient adsorbent for dye-contaminated water.

### 3.5. Regeneration performance of MB and MO by XG/CMC/silica hybrid

**Fig. 9:**
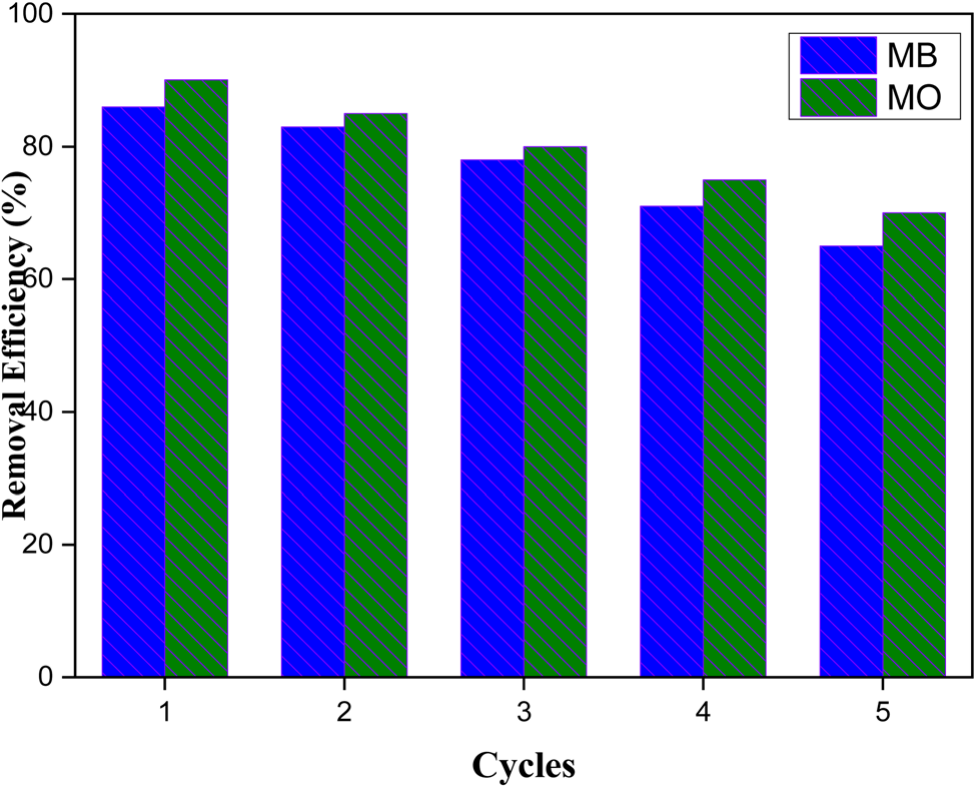
Reusability of XG/CMC/silica for the adsorption of MO and MB.

Reusability is a critical parameter in evaluating the practical applicability and economic viability of adsorbent materials, particularly for environmental remediation and wastewater treatment.^37^ The XG/CMC/silica hybrid demonstrated promising reusability performance, as depicted in Fig. 9. The adsorbent was subjected to five consecutive adsorption–desorption cycles using (MB) and (MO) as pollutants model. Even after five reused cycles, the hybrid retained considerable adsorption capacity, with efficiencies of approximately 65 mg g^−1^ for MB and 70 mg g^−1^ for MO after the 5th cycle. This relatively stable performance indicates that the hybrid matrix maintains its structural integrity and active binding sites over multiple cycles, with minimal degradation or leaching of functional groups.

### 3.6. Adsorption mechanism of MB and MO by xanthan gum/CMC/silica hybrid

**Fig. 10:**
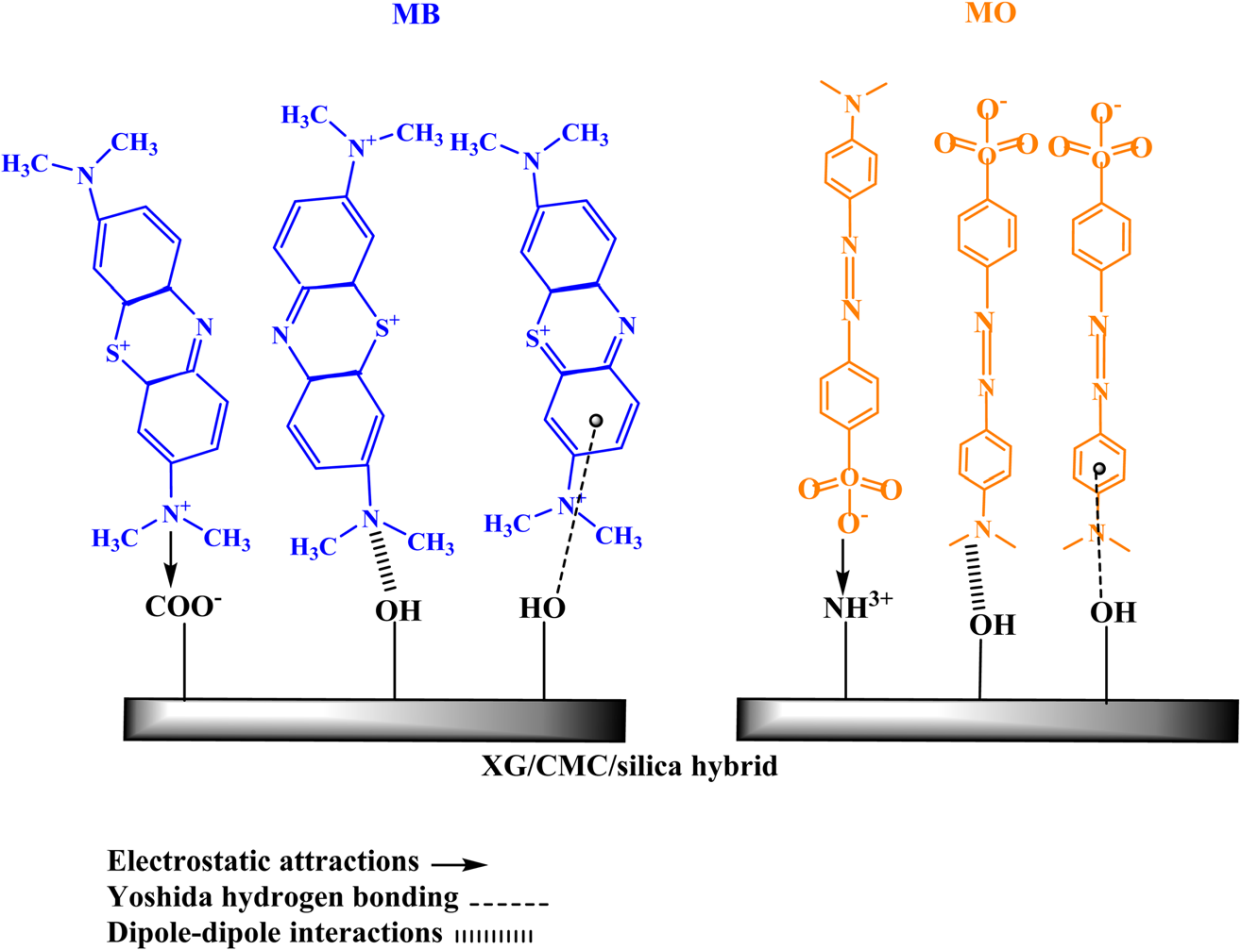
Schematic illustration of adsorption mechanisms of MB and MO onto XG/CMC/silica hybrid surface under acidic conditions.

In the case of the adsorption both MB and MO on the XG/CMC/silica hybrid, monolayer chemical adsorption mechanism is presumed due to electrostatic attraction and hydrogen bonding (Fig. 10). Due to deprotonation of the carboxylate groups (–COO^−^) of XG and CMC, the surface of XG/CMC/silica hybrid shows negative charge in alkaline aqueous solution. Since MB is a cationic dye bearing a positively charged quaternary ammonium group, it will tend to be adsorbed on the negatively charged adsorbent through electrostatic attraction. The electrostatic interaction may be a significant factor for the adsorption of MB.^38^ Furthermore, the hybrid material is rich in hydroxyl (–OH) groups, which can form dipole–dipole hydrogen bonds with the lone electron pairs on the nitrogen atoms of MB. These secondary interactions reinforce the adsorption process and contribute to the monolayer chemisorption behavior observed in equilibrium isotherm studies. Additionally, specific Yoshida-type hydrogen bonding may contribute between hydroxyl groups on the hybrid surface and aromatic structures in MB, further stabilizing the dye–adsorbent interaction. In acidic aqueous environments, the amino groups (–NH_2_) introduced by aminopropyl trimethoxysilane undergo protonation, converting to –NH_3_^+^. This confers a positive surface charge to the XG/CMC/silica hybrid. MO, an anionic azo dye, carries a negatively charged sulfonate group (–SO_3_^−^) under acidic conditions. Consequently, strong electrostatic attractions arise between the protonated hybrid surface and the anionic dye molecules. As with MB, hydroxyl groups on the hybrid surface may participate in dipole–dipole interactions with the electron-rich nitrogen atoms in MO. Moreover, potential Yoshida hydrogen bonding between the hydroxyl functionalities and the azo or aromatic groups in MO may play a role in stabilizing the adsorbed species.^39^ The isotherm analysis suggests that MO also follows a monolayer adsorption pattern, characteristic of chemical adsorption, with favorable energetics under acidic conditions.

To further support the proposed adsorption mechanism, FTIR spectra of the XG/CMC/silica hybrid before and after adsorption of MB and MO were recorded and are presented in Fig. S2. The pristine XG/CMC/silica hybrid exhibited the characteristic bands of the polysaccharide/silica network, including the broad –OH stretching band, C–H stretching vibrations, C=O/COO^−^ stretching, C–O–C/C–O vibrations, and Si–O–Si bands, confirming the coexistence of organic and inorganic functional domains. After dye adsorption, changes in the intensity of the –OH, C=O/COO^−^, and Si–O–Si/C–O bands were observed, indicating that these groups participated in dye binding. For MB adsorption, the interaction is mainly attributed to electrostatic attraction between cationic MB and negatively charged carboxylate/silanol groups, together with hydrogen bonding involving hydroxyl groups. For MO adsorption, the interaction is mainly associated with electrostatic attraction between anionic MO and protonated amino groups under acidic conditions, supported by hydrogen bonding with hydroxyl and silanol groups. Therefore, the FTIR results confirm that electrostatic interactions and hydrogen bonding are the main driving forces for MB and MO adsorption onto the XG/CMC/silica hybrid.

Table 4 shows the comparisons of adsorption capacities of the hybrid material with the other polysaccharide-based adsorbents that have been reported. The hybrid prepared exhibits adsorption capacities of 116 mg g^−1^ for MB and 107 mg g^−1^ for MO, which is comparable to the most effective bio-based adsorbents for dye removal. The excellent adsorption performance is attributed to the combination of biopolymeric matrix and silica, which contributes to the abundant surface functional groups and increases accessibility to adsorption sites. Furthermore, the presence of negatively charged carboxyl groups and positively charged amino groups on the hybrid simultaneously provides robust interactions for both cationic and anionic dyes. Therefore, the hybrid XG/CMC/silica could serve as a broad and versatile adsorbent for mixed dye wastewater.

**Table 4:** Maximum adsorption capacities of XG/CMC/silica hybrid compared with polysaccharides-based materials

Adsorbent	Maximum adsorption (mg g^−1^)	Adsorbate	References
Chitosan/Fe_3_O_4_ nanocomposites	758	MB	40
Chitosan microsphere	207	MO	41
Cellulose/silk/calcium phosphate	172	MB	42
Karay gum/chitosan/Ag nanocomposite	91	MB	43
Karaya gum/montmorillonite nanocomposite	156, 150, 138 and 129	MB, toluidine blue, crystal violet and azure B	44
Protonated cross-linked chitosan	89	MO	45
Oxidized cellulose	62	MB	46
Crosslinked carboxymethyl cellulose-based membrane	128	MB	47
XG/CMC/silica hybrid	116 and 107	MB and MO	The current study

## 4 Conclusion

In this study, the XG/CMC/silica hybrid adsorbent was successfully prepared by sol–gel method with APTES as the silica coupling agent. FTIR spectra indicated the preservation of specific functional groups in the polysaccharide along with the presence of Si–O–Si bonds, while XRD results showed that the integration of silica reduced the crystallinity of the XG/CMC matrix to yield an amorphous hybrid organic–inorganic composite. From SEM and EDX, the formation of porous structure and uniform dispersion of silicon in the composite was clearly shown, and the result from TGA suggested that the introduction of silica improved the thermal stability and the char yield of the material. Both MB and MO could be absorbed quite well on the hybrid adsorbent and the highest Langmuir capacities of the hybrid are 116 mg g^−1^ for MB and 107 mg g^−1^ for MO. The pH of solution played a significant role in this study since carboxyl groups is benefit to adsorpt cation MB and protonated amino group contribute to adsorpt anionic MO in acidic media. The adsorption kinetics could well fit the pseudo first-order model and equilibrium isotherms fitted Langmuir model suggesting monolayer adsorption occurred on active sites with easily accessible surfaces. The spontaneous and endothermic nature of the adsorption was indicated by thermodynamics. Furthermore, this hybrid still achieved favorable adsorption efficiencies even after 5 regeneration cycles, which indicate the excellent reusability of the adsorbent. In summary, the XG/CMC/silica hybrid is a promising, efficient, renewable bio-based material for dye removal. Future work should be directed towards studying its adsorption efficiency towards actual industry wastewater, mixtures of dyes and highly saline solutions.

## Author contributions

Ehab S. Gad: writing – review & editing, data curation. Tayel A. Al Hujran: formal analysis, investigation, writing – review & editing. Saad Alrashdi: writing – review & editing. Mohammed A. Amin: writing – review & editing. Medhat E. Owda, methodology, writing – review & editing, data curation, Yousef A. Bin Jardan: formal analysis, investigation, review & editing.

## Conflicts of interest

This paper was written by authors without personal conflicts of interest.

## Supplementary Material

RA-016-D6RA05243F-s001

## Data Availability

All data generated or analyzed during this study are included in this published article. Supplementary information (SI): FTIR spectra of native and deacetylated xanthan gum (Fig. S1), as well as FTIR spectra of the XG/CMC/silica hybrid before and after methylene blue and methyl orange adsorption and after five adsorption cycles (Fig. S2). These results support the xanthan gum deacetylation process, the proposed dye-adsorption mechanism, and the structural stability of the hybrid adsorbent after regeneration. See DOI: https://doi.org/10.1039/d6ra05243f.
