# Supplementary material for: Sol–gel engineered xanthan gum/carboxymethyl cellulose/silica hybrid adsorbent for efficient removal of cationic and anionic dyes
Source: RSC Adv. 2026 Jul 15. Online ahead of print. doi: 10.1039/d6ra05243f (PMC13372137; doi:10.1039/d6ra05243f)
Supplement: RA-OLF-D6RA05243F-s001 [file RA-OLF-D6RA05243F-s001.pdf]

Supplementary Information (SI) for RSC Advances.
This journal is © The Royal Society of Chemistry 2026

Supporting Information

Sol–Gel Engineered Xanthan Gum/Carboxymethyl Cellulose/Silica Hybrid Adsorbent for Efficient Removal of Cationic and Anionic Dyes

Ehab S. Gad [a], Tayel A. Al Hujran[b], Saad Alrashdi[a], Mohammed A. Amin[c], Medhat E. Owda*[d], Yousef A. Bin Jardan[e]

[a]Department of Chemistry, College of Science, Jouf University, Sakaka 72341, Aljouf, Saudi Arabia.

[b] Department of Pharmaceutical Chemistry, Faculty of Pharmacy, Mutah University, Jordan.

[c] Department of Pharmaceutics, College of Pharmacy, Qassim University, Qassim 51452, Saudi Arabia.

[d]Department of Chemistry, Faculty of Science (boys), Al-Azhar University, 11884 Nasr City, Cairo, Egypt.

[e] Department of Pharmaceutics, College of Pharmacy, King Saud University, Riyadh, Saudi Arabia.

Corresponding author: Medhat E. Owda(*medhatowda@azhar.edu.eg*)

The FTIR spectra of XG and D-XG exhibited very similar overall polysaccharide features, indicating that the alkaline treatment preserved the xanthan backbone. A reduction in the intensity of the carbonyl region around 1730–1740 cm^{-1} was observed after alkaline treatment, suggesting partial removal of acetyl groups from xanthan gum. The preservation of the characteristic carboxylate and glycosidic bands demonstrates that deacetylation occurred without significant degradation of the polymer backbone.

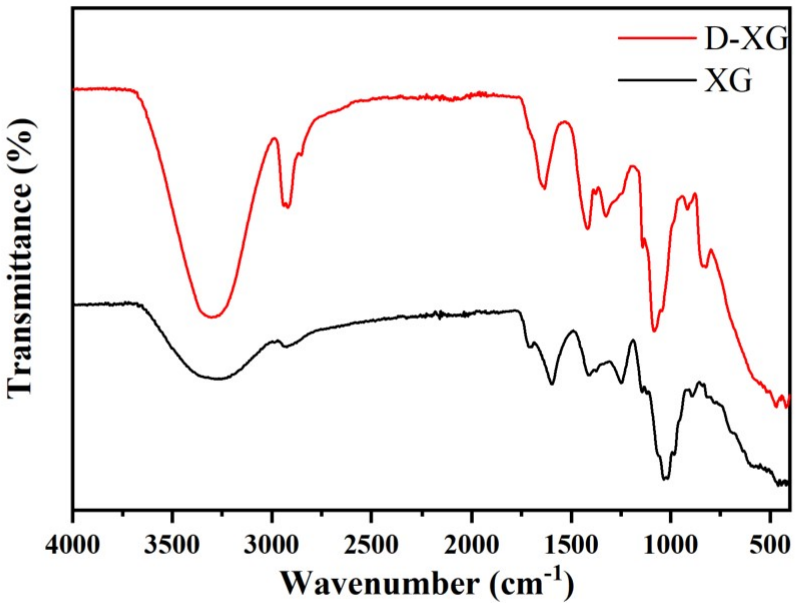

Figure S1. FTIR spectra of native xanthan gum (XG) and deacetylated xanthan gum (D-XG).

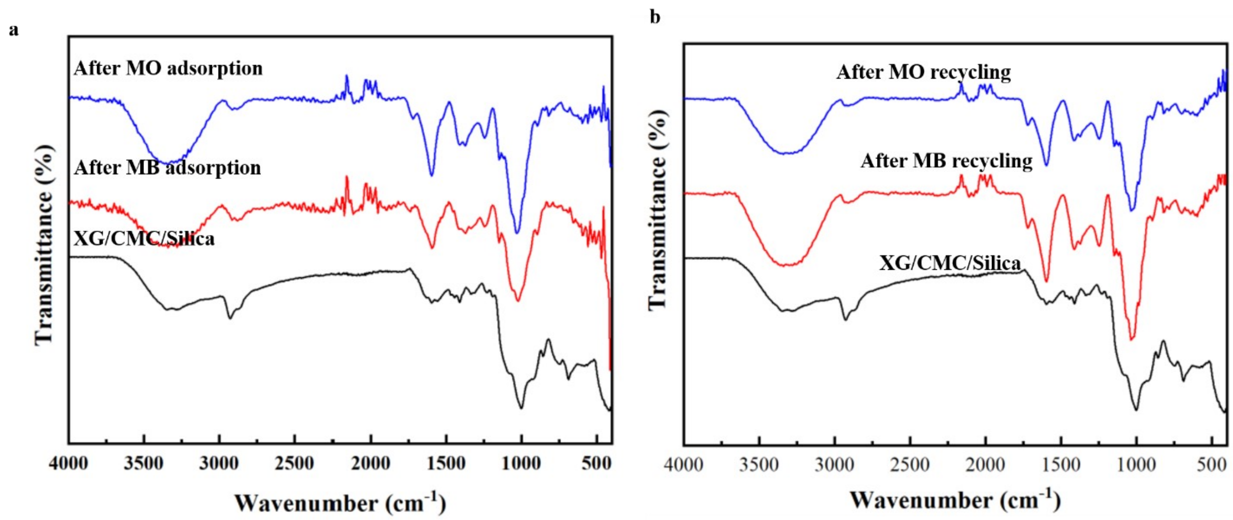

Figure S2. FTIR spectra of XG/CMC/silica before and after MB/MO adsorption (a) and after recycling (b).
